# Hmrbase: a database of hormones and their receptors

**DOI:** 10.1186/1471-2164-10-307

**Published:** 2009-07-09

**Authors:** Mamoon Rashid, Deepak Singla, Arun Sharma, Manish Kumar, Gajendra PS Raghava

**Affiliations:** 1 Bioinformatics Centre, Institute of Microbial Technology, Sector 39A, Chandigarh-160036, India

## Abstract

**Background:**

Hormones are signaling molecules that play vital roles in various life processes, like growth and differentiation, physiology, and reproduction. These molecules are mostly secreted by endocrine glands, and transported to target organs through the bloodstream. Deficient, or excessive, levels of hormones are associated with several diseases such as cancer, osteoporosis, diabetes etc. Thus, it is important to collect and compile information about hormones and their receptors.

**Description:**

This manuscript describes a database called Hmrbase which has been developed for managing information about hormones and their receptors. It is a highly curated database for which information has been collected from the literature and the public databases. The current version of Hmrbase contains comprehensive information about ~2000 hormones, e.g., about their function, source organism, receptors, mature sequences, structures etc. Hmrbase also contains information about ~3000 hormone receptors, in terms of amino acid sequences, subcellular localizations, ligands, and post-translational modifications etc. One of the major features of this database is that it provides data about ~4100 hormone-receptor pairs. A number of online tools have been integrated into the database, to provide the facilities like keyword search, structure-based search, mapping of a given peptide(s) on the hormone/receptor sequence, sequence similarity search. This database also provides a number of external links to other resources/databases in order to help in the retrieving of further related information.

**Conclusion:**

Owing to the high impact of endocrine research in the biomedical sciences, the Hmrbase could become a leading data portal for researchers. The salient features of Hmrbase are hormone-receptor pair-related information, mapping of peptide stretches on the protein sequences of hormones and receptors, Pfam domain annotations, categorical browsing options, online data submission, DrugPedia linkage etc. Hmrbase is available online for public from .

## Background

According to the Medical Subject Heading (MeSH), hormones are defined as chemical substances having a specific regulatory effect on the activity of a certain organ or organs, although the classical definition of hormones limits them to the domain of chemical signaling molecules produced by endocrine glands and secreted directly into the bloodstream. Hormones travel through the blood to distant tissues and organs, where they can bind to specific cell sites called receptors. By binding to receptors, hormones trigger various responses in the tissues/cells containing cognate receptors [1,2]. On the basis of their chemical natures, hormones are broadly classified into protein/peptide hormones (genome-encoded) and non-peptide hormones (non-genome-encoded). Hormone-receptor interactions are amongst the most important ligand-receptor type of interactions in biological systems. The living multicellular entity depends on complex communication networks for its survival. Hormones, acting as chemical messengers, are the postmen of endocrine machinery. The endocrine system focuses on ligand-receptor interactions to play a critical role in growth and development of multicellular eukaryotes [3,4]. The data flow in this area of biological science is rapid and vast. Therefore, collection and compilation of information about these interactions, and underlying molecules (hormones and receptors), will be useful.

In recent years, efforts have been made to collect and organize receptors (like GPCRDB, ORDB, NuReBase and GRIS) [5-8]. These databases deal with different classes of receptors in biological system; for example, GPCRDB/GRIS/ORDB for G-protein coupled receptors (GPCRs) and NuReBase for nuclear hormone receptors. Various type of databases; for example SwePep [9] for endogenous peptides and PepBank [10] for peptides collected from literature using text mining tools, came into existence recently. There are a few databases which maintain information about ligands and their receptors like PRRDB [11], GLIDA [12], and EndoNet [13]. PRRDB, an immunological database, provides information regarding Pattern Recognition Receptors and their ligands. GLIDA is developed with possible implications in chemical genomic research and GPCR-related drug discovery, whereas EndoNet is an information resource about intercellular regulatory communication. Though existing databases provide important information, there is lack of a comprehensive resource on hormones and their receptors.

In order to complement existing databases in the field, and to understand hormones and their interaction with receptors, we have developed a database called Hmrbase. This database provides comprehensive information about hormones and receptors. Various data fields like hormone precursor, subcellular localization, post-translational modification, taxonomy, source organism, function, description, tissue specificity, molecular weight, similarity to other proteins, and mapping of hormone peptide on its corresponding precursor etc. have been included for peptide hormones and their receptor. For non-peptide hormones, the data fields consist of their names, molecular weights and molecular formulae, IUPAC names, canonical and isomeric smile formulae, melting points, LogP values, water solubility, and their corresponding receptors etc. Various co-ordinate files such as PDB, SDF, and MOL files are available for download. Structure visualization tools such as Advance Chemistry Development (ACD) structure drawing applet [14] (for 2-D visualization) and Jmol applet [15] (for 3-D visualization) have been embedded in Hmrbase. Links to neighbors (external links) like Swiss-Prot [16], PDB [17], NCBI Gene Database [18], Pfam [19], PubChem [20], KEGG [21], HMDB [22], DrugBank [23], and DrugPedia [24] have been incorporated in Hmrbase to make it a complete system. Moreover, hormones and receptors entries are linked to their corresponding receptors and hormones, respectively. Sequence similarity search, peptide mapping and domain search tool, in case of protein hormone and receptor, facilitates the extraction of useful information. In addition to text search, a structural similarity-based search option for non-peptide hormones supports the search algorithm. Thus, Hmrbase provides both comprehensive and easy-to-use information related to hormones and their receptors.

## Construction and content

For collection of peptide hormones, we have extensively searched Swiss-Prot, other databases and the related literature. Initially, we started searching through Sequence Retrieval System (SRS) of Swiss-Prot with a keyword "hormone" against the "Description" field with wild card. Then we exploited GPCRDB for hormone receptor. GPCR class A comprises hormone receptors such as serotonin, cholecystokinin, melanocortin, prolactin, somatostatin, vasopressin, adrenomedullin, melanin etc. GPCR class B comprises calcitonin, glucagons, diurectic, parathyroid, secretin hormone receptors. Regarding collection of non-peptide hormones we searched various databases like PubChem, Human Metabolome Database (HMDB), and EndoNet. The corresponding receptors were taken from PubChem, literature databases like PubMed, EndoNet, DrugBank, NucleaRDB [25], and Swiss-Prot. Detailed information about data collection and manual curation has been included in the additional file 1.

This database consists of extensively manually curated information about peptide hormones, non-peptide hormones, and their receptors. The statistics of the data has been given in Table 1 and Table 2. Following is the brief description of data available at Hmrbase.

**Table 1:** Hmrbase data types and their corresponding numbers

**Data types**		**Number of entries**	**Total**
Hormones	Peptide/protein	1585	1955
		
	Non-peptide	370	

Receptors (for)	Peptide hormone	828	2996
		
	Non-peptide hormone	2168	

Hormone-Receptor Pair	Peptide hormone-Receptor	569	4121
		
	Non-peptide hormone-Receptor	3552	

**Table 2:** Some of the biological taxa with corresponding number of entries in Hmrbase database

**Important taxa**	**Number of entries in database**	
	
	**Hormone (%)**	**Receptor (%)**
Chordate	1362 (69.6)	2309 (77.06)

Arthropoda	198 (10.1)	207 (6.90)

Mollusca	19 (0.97)	33 (1.10)

Metatheria	29 (1.48)	15 (0.50)

Eutheria	753 (38.51)	1391 (46.42)

Primates	170 (8.69)	478 (15.95)

Rodents	227 (11.61)	535 (17.85)

Cetartiodactyla (whales, hippos, ruminants, pigs, camels)	214 (10.94)	239 (7.97)

New world monkeys	13 (0.66)	49 (1.63)

Old world monkeys	44 (2.25)	69 (2.30)

Gorilla	5 (0.25)	3 (0.10)

Chimpanzee	14 (0.71)	14 (0.46)

Human	84 (4.29)	310 (10.34)

Values in parentheses are the percentage of hormones or receptors for a particular taxon included in Hmrbase database

### Peptide hormones (PH)

Different PHs from various taxonomical classes have been collected and compiled. At present the database encapsulates 1585 PHs. A pool of information has been supplied with each hormone entry. The type of information has been explained under "data structure" heading.

### Non-Peptide hormones (NPH)

These are basically small chemicals and play an important role in signal transduction pathways regulating complex networks of gene expression. A total of 370 such molecules have been compiled in Hmrbase.

### Receptors for peptide hormones (RPH)

Altogether, there are 828 receptor entries for peptide hormones. Mostly, these are G-protein coupled receptors (GPCR) on the membranes of cell surfaces, which sense external stimuli (in the form of ligands) to transduce the information to intracellular region.

### Receptors for non-peptide hormones (RNH)

Receptors for non-peptide hormones are mainly ligand-activated nuclear transcription factors. These are actively involved in alterations of gene expression which, in turn, regulate the normal physiology of an organism. A total of 2168 RNH have been maintained in Hmrbase.

### Hormone-receptor pair

To understand the functional diversity and the mode of action of any hormone, information about its receptor is essential. Approximately, 4121 hormone-receptor functional interactions have been incorporated in Hmrbase.

## Utility

### Web Tools

Apart from the collection of hormone molecules and their receptors, a wide variety of information can be generated using the online software/tools provided with Hmrbase database. Following are the main web tools provided with the Hmrbase database:

#### Keyword Search

There are separate search pages for hormones and receptors. Both are almost identical in architecture except for few data fields. Searching can be performed on any field separately, or on all fields simultaneously, using a specific keyword, to retrieve data from the database. The search option is restricted to almost all the data fields available in the database. User can define which search results are to be displayed.

#### Structural Similarity Search

**Figure 1:**
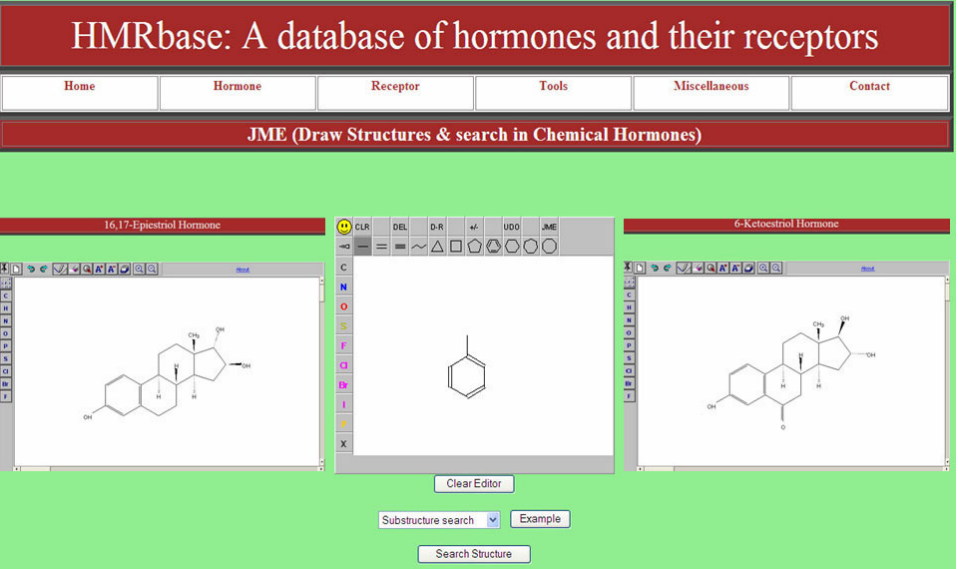
**An example of structural similarity based search of Hmrbase entries**. An example of structure based search of Hmrbase entries where a phenyl group was used as a query substructure. Structure search resulted in two entries namely 16, 17-epiestriol and 6-keto estriol hormones.

Hmrbase also provides a tool to search for entries related to non-peptide hormones, based on their structural similarities. JAVA Molecular Editor (JME) [26] along with JC search tool [27] facilitates the structure based searching of the Hmrbase entries. Structures drawn in the JME editor are used by JC search tool to search for similar structures in the database. Users can also select one of the different search types e.g. substructure search, exact search, superstructure search etc. Figure 1 illustrates an example of structure based search of Hmrbase entries where a phenyl group was used as a query substructure. Structure search resulted in two entries namely 16, 17-epiestriol and 6-keto estriol hormones.

#### 2-D and 3-D structure visualization tools

**Figure 2:**
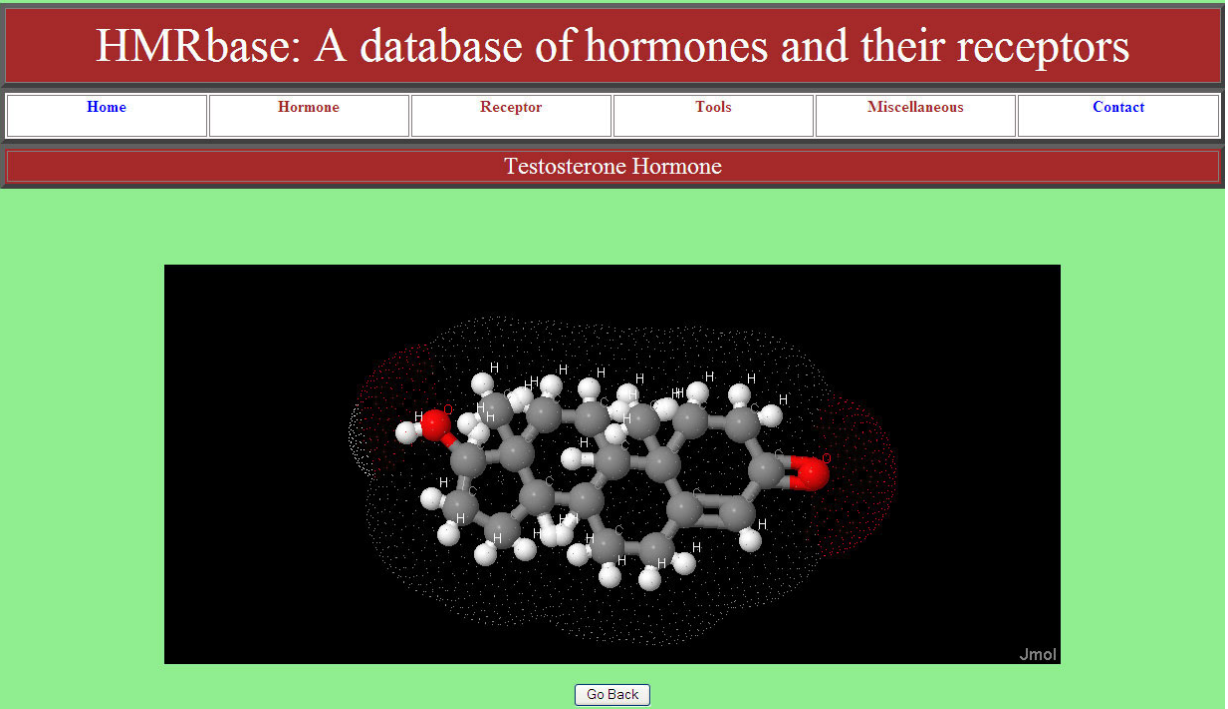
**Structure visualization by Jmol**. Structure of testosterone hormone as visualized in Jmol, showing different labels of atoms; white balls are hydrogen atom, red oxygen, and gray are carbon atoms.

For non-peptide hormones, Hmrbase is facilitated by structure display tools. Jmol and Advanced Chemistry Development (ACD) structure drawing applets have been implemented, to show 3-D and 2-D structures, respectively. Figure 2 illustrates a 3-D structure of testosterone hormone displayed using the Jmol applet embedded in Hmrbase.

#### DrugPedia

**Figure 3:**
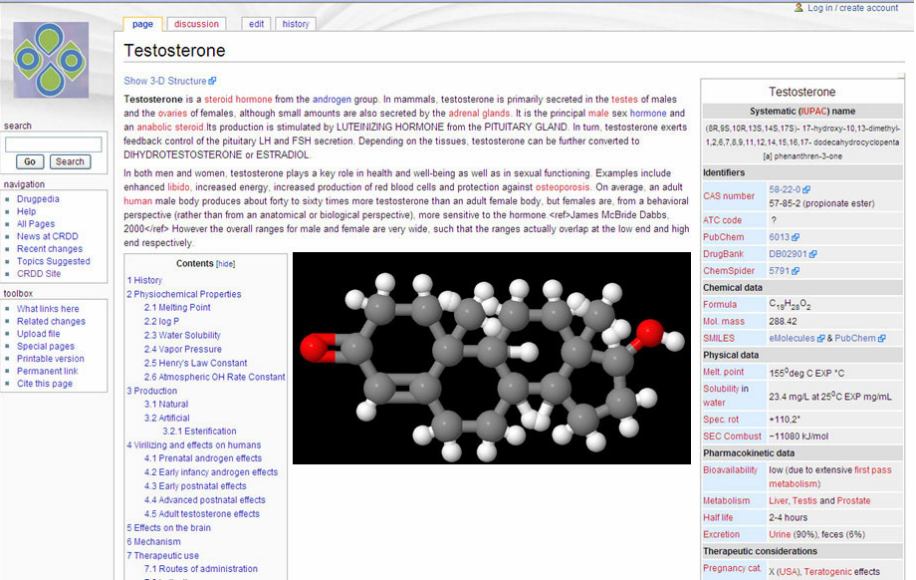
**A typical DrugPedia page for testosterone hormone**. Figure shows a typical DrugPedia page for testosterone hormone. Top right corner contains link for new account creation and login. To edit, discuss, or see history of a particular page, links are embedded on top left of the page.

Hmrbase provides a DrugPedia link corresponding to each entry. Data stored in the Hmrbase platform follow a definite pattern. Therefore, to provide more flexibility to different kinds of data, a DrugPedia link would be beneficial in which users can update or add any relevant information. The important and relevant information from the DrugPedia page may be included in the main database frame after validation during the updation of Hmrbase. Figure 3 shows a typical DrugPedia page for testosterone hormone.

#### Sequence Similarity Search

A customized BLAST [28] tool has been made available which searches a user-defined query against the sequence of the hormone, or receptor, or both. It may be useful in characterization of orphan receptors and fishing out of homologous sequences from the database, based on sequence similarity.

#### Peptide Mapping

Users can map active subsequences or stretches of amino acids on the hormone and/or receptor protein sequences. This will add to the information regarding the distribution of functional stretches of amino acid over the entire hormone and/or the receptor sequences present in the database. Such a type of mapping might be useful in understanding the functional diversities of biologically active peptides. Peptide mapping in Hmrbase is simply implemented using "exact string search", which searches user defined queries for peptides in hormone/receptor protein sequences.

#### Pfam Domain Search

A group of functionally similar sequences may represent common peptide domain(s). This concept underlies the domain-wise classifications of protein families and sub-families. Therefore, the types of domain(s) present in a hormone, and in receptor protein sequences, determine their classification. Thus, the Pfam domain search algorithm has been embedded into the Hmrbase platform. It has been observed that some domains are highly frequent and some are rare amongst Hmrbase entries. All the unique domains from peptide hormone and receptor entries along with their frequency distribution have been shown in Table 3.

**Table 3:** Unique Pfam domains and their occurrence among Hmrbase entries

	**Peptide Hormone**		**Receptor**	
	
**Serial no**.	**Pfam Domain Name**	**Frequency**	**Pfam Domain Name**	**Frequency**
1.	Insulin	253	zf-C4	1593

2.	Hormone_1	170	Hormone_recep	1459

3.	ACTH_domain	166	7tm_1	824

4.	Op_neuropeptide	156	7tm_2	129

5.	Hormone_2	144	HRM	119

6.	Gastrin	96	LRR_1	42

7.	Cys_knot	78	fn3	34

8.	Somatostatin	55	DUF1856	13

9.	Hormone_6	54	EGF	7

10.	Thymosin	46	EGF_CA	7

11.	Crust_neurohorm	45	Laminin_G_2	6

12.	Hormone_3	33	Laminin_G_1	6

13.	CRF	24	Furin-like	4

14.	Motilin_assoc	20	Recep_L_domain	4

15.	Motilin_ghrelin	20	Ldl_recept_a	4

16.	ELH	18	Calsequestrin	1

17.	Bombesin	11	Homeobox	1

18.	TRH	10	Thioredoxin	1

19.	Hormone_5	9		

20.	Adipokin_hormo	8		

21.	Eclosion	3		

22.	Crust_neuro_H	2		

Frequency of a domain is the number of entries in Hmrbase database harboring that domain.

### Data Structure

**Figure 4:**
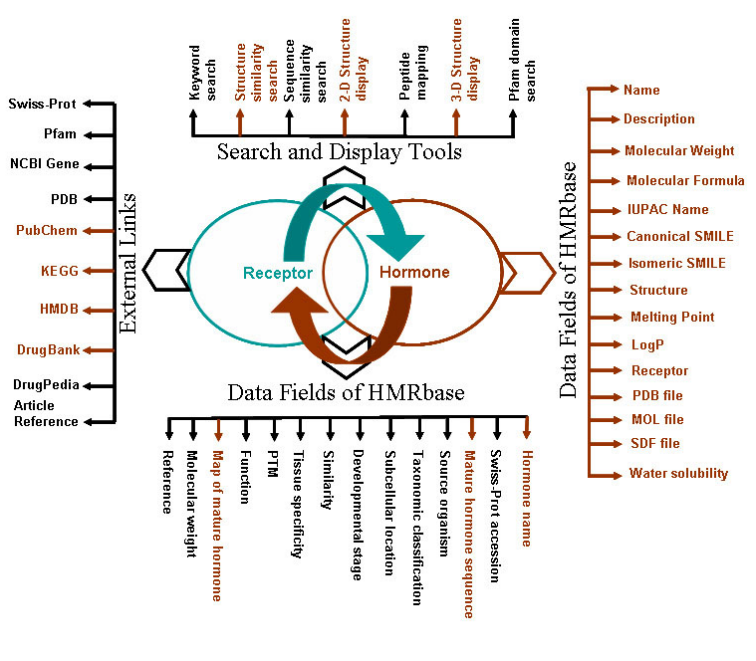
**Data structure of Hmrbase**. Data types for hormone and receptor tables have been shown in different color schemes; black colored data types/tools are common for hormones as well as receptors, brown colored are exclusively for hormones.

Several tables in relational fashion facilitate the data architecture of Hmrbase. There are three primary tables at the front, one each for peptide hormones, non-peptide hormones and receptors. The query from the user is replied to with the help of these three tables. The table for peptide hormones provides a 'series' of information like hormone name, active sequence, length of active sequence, mapping of active sequence on its precursor, related references, function, Swiss-Prot accession number, precursor/protein description, source organism, taxonomy, subcellular localization, developmental stage, similarity, tissue specificity, post-translational modification, length and molecular weight (in Dalton) of precursor/source. Each non-peptide hormone entry presents the name, description, IUPAC name, canonical and isomeric smile, melting point, LogP, water solubility, molecular weight, molecular formula, corresponding receptor, and external links to different databases such as PubChem, PDB, KEGG, HMDB, DrugBank etc. Each entry about a hormone is internally linked to its corresponding receptor entry in the receptor table. Moreover, each of the Hmrbase entries has been linked to DrugPedia (a Wikipedia for Drug Discovery), where users may contribute data and updated information. Further, FASTA sequences can be downloaded by clicking the link supplied by hormone and receptor tables. The table for receptors supplies more or less the same information as peptide hormone table. Bi-directional cross-linking has been provided between hormone entry and its corresponding receptor entry. Figure 4 illustrates the whole data structure of Hmrbase.

### Data Flow

**Figure 5:**
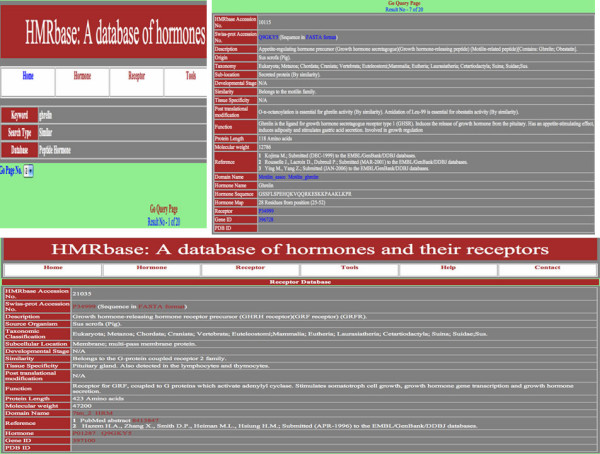
**Data flow in Hmrbase**. Hmrbase hormone search result page using keyword "ghrelin" as description, a total of 20 entries were found (top left panel); out of 20 hormone entries, one complete entry has been shown for Swiss-Prot accession number Q9GKY5 and its receptor counterpart is P34999 (top right panel); by clicking the link for P34999 a complete entry page for receptor P34999 appears and among its ligand counterparts is Q9GKY5 (bottom panel).

There are separate search pages to query hormone, or receptor, entries. Keyword search options operate on a variety of data types like hormone name, description, developmental stage, source organism, functions etc. Each hormone entry is linked to its corresponding receptors (if available). In a similar way the receptor search result page leads towards their corresponding ligand(s). This scheme of data flow is depicted in Figure 5. The keyword "ghrelin" fetched a total of 20 entries (Figure 5, top left panel) from hormone table of Hmrbase. One of the entries out of 20 was for Swiss-Prot accession number Q9GKY5, showing corresponding receptor entry (P34999) along with other data fields (Figure 5, top right panel). Figure 5 (bottom panel) showed complete page for P34999 from receptor table. The Hmrbase entries provide links to many external databases such as Swiss-Prot, NCBI Gene, PDB, Pfam etc. Moreover, each and every entry was also linked to DrugPedia, where one can contribute recent or updated information.

### Web Interface and Application

Simple HTML and CSS technologies have been used to build the static web interface. MySQL, an object-relational Database Management System (RDBMS), works at the backend. Server-side scripting makes use of PHP. The whole software system runs on IBM SAS x3800 machine under RedHat Enterprise Linux 5 environment using Apache httpd server. PHP and MySQL combination is quite efficient and powerful for database management.

## Discussion and conclusion

Hmrbase is a comprehensive information resource about hormones and their receptors. Ligand-receptor interactions have been elucidated in a bi-directional manner. Users can start from searching hormone entry (ies) and end up in their corresponding receptor (s); and vice-versa. The database will fulfill the requirements of theoretical as well as clinical endocrinologists. Data structure of Hmrbase is quite simple and convenient for general users. The mature hormone sequence is mapped on its precursor protein sequence in order to define the functional modes of hormones. Furthermore, this information can be exploited by experimental scientists to design better ligands for a particular receptor or for studying binding affinity of hormones to their corresponding receptors. Protein domains have been inferred as the basic building blocks of protein interactions [29]. Therefore, to derive the Pfam domains distribution among the entries of Hmrbase, a domain search facility has been embedded.

Furthermore, a collection of non-peptide hormone molecules along with various operational tools such as ACD/Structure drawing applet, Jmol, and JC Search with JME editor facilitates the completeness of the database.

Hopefully this customized database will expand quantitatively as well as qualitatively in coming days to cover the annotation gaps such as orphan receptors and probably any novel hormone molecule.

## Application of Hmrbase

Hmrbase presents data in a sophisticated way. Apart from text search, several browsing options facilitate the retrieval of important datasets, such as entries for a particular organism or hormone name or specific domain or domain combinations etc. Moreover, hormone-receptor and receptor-hormone pairs have been presented to infer the range of action of a particular hormone or receptor. Different types of structure search algorithm such as substructure, exact, superstructure search would help in compiling the set of entries containing a particular functional group or moiety. Moreover, each entry of Hmrbase has been linked to DrugPedia, which would serve as a complement to Hmrbase entries. Any new or updated information posted on DrugPedia would be included in Hmrbase database after validation. Thus Hmrbase would be a comprehensive and stable system for biomedical researchers and bioinformatician.

## Limitations and future prospects

Several new data types such as pharmacological data are being collected to incorporate into Hmrbase. The major limitation of this resource is the lack of a fully automated database populating system. Nevertheless, we have devised different models for updation of Hmrbase at a period of every three month (see Additional file 1).

## Availability and requirements

Hmrbase is available at: . To access all features of Hmrbase database to its optimum level, javascript and Java Runtime Environment (JRE) plugin must be enabled.

## Authors' contributions

MR collected and compiled the data from literature and public databases. MR made the structure of the database. DS collected non-peptide hormone molecules and their nuclear receptor counterparts. MK and MR incorporated various amino acid sequence manipulative tools in Hmrbase. Web interface was designed by AS and MR. AS contributed in converting Perl scripts into PHP and PgSQL tables into MySQL tables originally made by MR and MK. AS and DS incorporated tools for non-peptide hormone manipulation. GPSR conceived the project, coordinated it and refined the manuscript drafted by MR.

## Supplementary Material

Additional file 1 **Supplementary material**. It consists of detail strategy of data collection in Hmrbase and the updation schemes. Click here for file
