# Supplementary material for: Hmrbase: a database of hormones and their receptors
Source: BMC Genomics. 2009 Jul 9;10:307. doi: 10.1186/1471-2164-10-307 (PMC2720991; doi:10.1186/1471-2164-10-307)
Supplement: Additional file 1 — Supplementary material. It consists of detail strategy of data collection in Hmrbase and the updation schemes. [file 1471-2164-10-307-S1.pdf]

# Supplementary material

# Strategy followed in data collection

## Hormone

Chemical substances having a specific regulatory effect on the activity of a certain organ or organs are termed as hormones. The term was originally applied to substances secreted by various ENDOCRINE GLANDS and transported in the bloodstream to the target organs. It is sometimes extended to include those substances that are not produced by the endocrine glands but that have similar effects.

During collection of entries of HMRbase the above definition of hormone was kept in mind.

## Collection of Peptide hormones and their receptor

<u>Peptide Hormones (primary search):</u> (I) First we searched word "hormone" in the field "Description" of Swiss-Prot using Sequence Retrieval System (SRS) interface and got total of 1122 entries; (II) Each entry was inspected manually and extract 630 hormones; (III) Full information about each hormone was extracted from literature.

<u>Peptide Hormone Receptors (primary search):</u> (I)Receptors corresponding to above peptide hormones were searched from Swiss-Prot, literature and Internet; ( II) We got 492 receptors corresponding to 630 hormones as receptor for each hormone is not reported in literature; (III) We extract 855 hormone receptors from GPCR class A (e.g., serotonin, cholecystokinin, melanocortin, prolactin, somatostatin, vasopressin, adrenomedullin, melanin) and GPCR class B (e.g. calcitonin, glucagons, diurectic, parathyroid, secretin hormone receptors) for database GPCRDB .

<u>Peptide Hormones and receptors (secondary search):</u> (I) Hormones corresponding to above 1347 (492 + 855) hormone receptors were searched in databases and literature; (II) We obtained 383 new hormones corresponding to these 1347 hormone receptors; (III) We also extract 84 insulin hormones

from literature; (IV) This way, we got a total of 1097 (630 + 383 + 84) hormone molecules (V) We searched literature again corresponding to above hormone peptides and got a total of 1546 hormone receptors.

Refinement of Data: We got 996 hormones and 1334 receptors after removing redundant entries. The manual inspection of 1342 hormone receptors revealed that 137 entries were not originally hormone receptors and were removed. We manually inspected each of the 996 hormone entries and found that some of the entries are hormone precursors containing more than one hormone. We further analysed these 996 entries, thus the number of individual hormone molecules increased to **1521**. The whole process of obtaining these hormones and their ligands were very tedious and accomplished with the help of perl scripts and manual inspection side-by-side. Moreover, we have also taken data from other databases (where sufficient information was available) like NUREBASE, GRIS etc.

## Collection of Non-peptide hormones and their receptor

Non-peptide hormones: We searched various databases like PubChem, Human Metabolome Database (HMDB), Endonet etc. A total of 370 non-peptide hormones were collected. The corresponding receptors were taken from PubChem, Endonet, DrugBank, NucleaRDB, and Swiss-Prot. The following table depicts the data types and their source of collection for non-peptide hormone-

| **Data Types** | **Source** |
|---|---|
| ID | Unique, Start from NPH101 |
| PubChem ID | PubChem, HMDB |
| Name | PubChem, HMDB |
| Molecular Weight | PubChem, HMDB |
| Formula | PubChem, HMDB |
| IUPAC Name | PubChem, HMDB |
| Canonical Smiles | PubChem, HMDB |
| Isomeric Smiles | PubChem, HMDB |
| PDB File | HMDB |
| SDF File | Pubchem,HMDB |

| | |
|---|---|
| MOL File | Pubchem,HMDB,Converted from ChemDraw |
| PDB ID | PubChem |
| KEGG ID | PubChem, HMDB |
| HMDB ID | |
| Melting Point | ChemD Plus |
| LogP | |
| Water Solubility | |
| Drug Bank ID | KEGG |
| Drugpedia ID | Link to DrugPedia by Name |
| Receptor | Endonet,Drug Bank, Literature |
| References | PubChem,HMDB,Endonet |
| Comments | Any comment for a hormone entry |

Thus, a total of 1955 hormones and 2996 hormone receptors have been included in HMRbase database.

## How to search the receptor for a particular hormone entry?

1. Swiss-Prot page related to a hormone molecule contains information about its receptor under various fields like "Function", "Similarity", "GO Molecular functions" etc.

2. Go to Drug bank ID search target field where name of receptor is given.

3. Go to Drug bank ID search for mechanism of action field where name of receptor is given.

4. Go to Endonet hormone entries then for receptor link where swiss prot ID is given.

5. Go to Pubmed and Google.com and search by keywords "**hormone name receptor"** or "**hormone name mechanism of action"**. A number of relevant articles or abstracts give information about receptor molecule.

6. Go to pubchem link for a hormone entry and click on **pharmacology** link in the literature classification.

## HMRbase Updation Scheme

We have devised four different models for updation of HMRbase. The first two models have been

implicated by database authors and rest two is managed by database users.

**Model 1:** We have written a Perl script for extracting data from PubMed. This program perform following task at regular interval; I) extract articles relevant to HMRbase from PubMed using E-UTILS (Entrez Programming Utilities); ii) these articles (pubmed ID) will be searched in Swiss-Prot database. Swiss-Prot entries and PubMed articles will be examined manually to update the HMRbase.

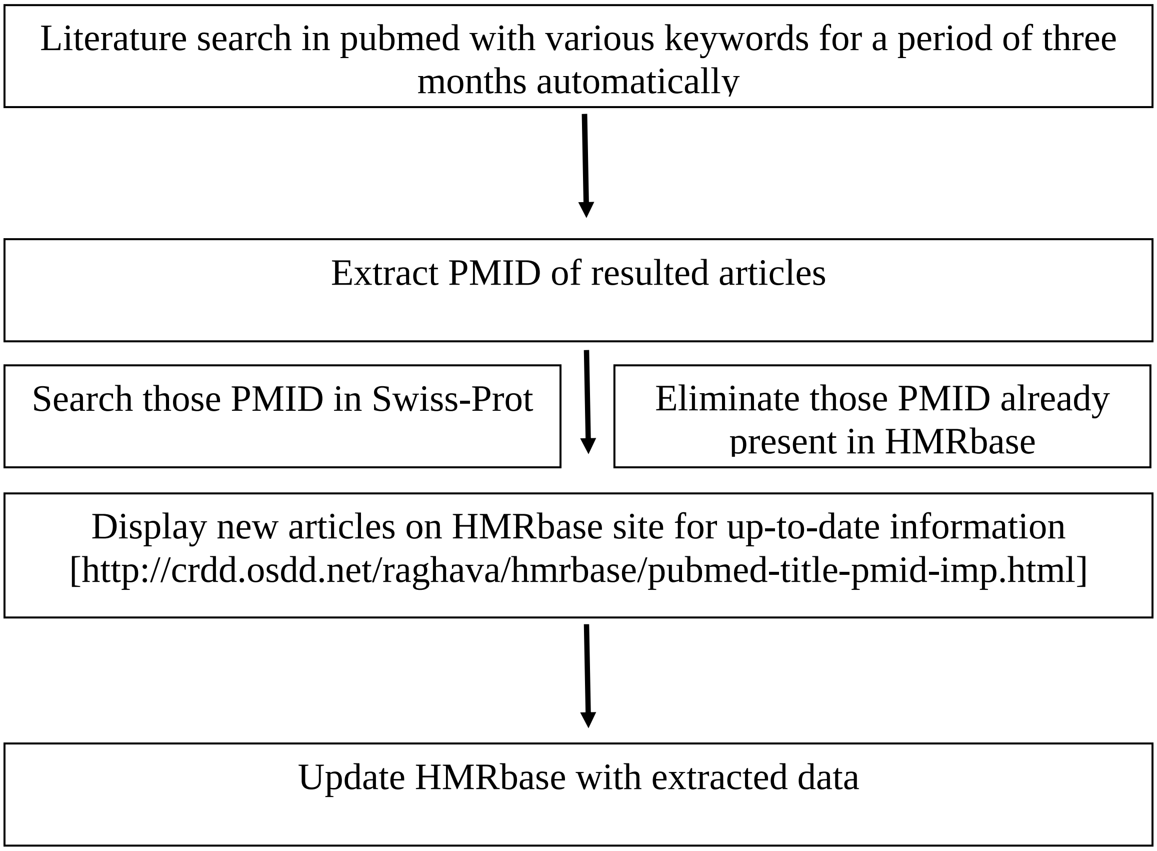


**Fig. 1. Model 1 scheme for HMRbase updation.**

**Model 2:** A program has been developed to extract information automatically from existing databases (e.g., Swiss-Prot) for latest entries. The articles referred in databases will be extracted from PubMed. Finally information from databases and from PubMed will be examined to update HMRbase.

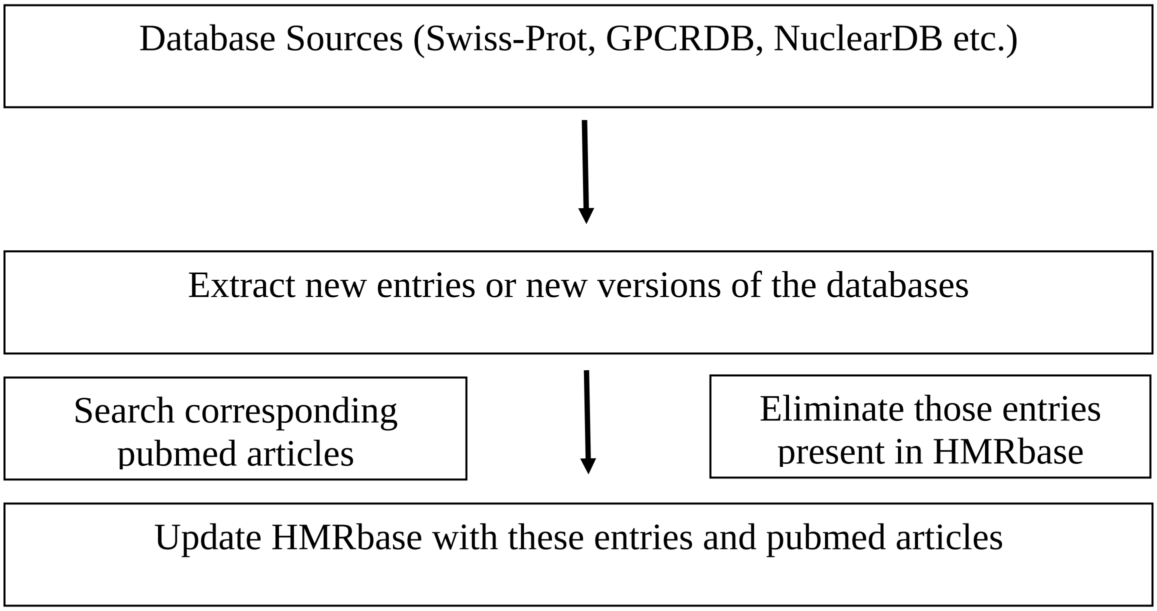


**Fig. 2. Model 2 scheme for HMRbase updation.**

**Model 3:** HMRbase has provided facility to submit data; user may add record to any table "Peptide-hormone", "Non-peptide hormone" and "Receptor". These records will be stored in temporary tables and will be examined by HMRbase administrator for its suitability.

**Model 4:** Each entry in HMRbase is also available in DrugPedia as a page. DrugPedia page have all information in HMRbase plus additional information. This DrugPedia is based on WikiPedia where any one can contribute to these articles/pages. We will monitor modifications in each page of DrugPedia and useful information will be used for updating HMRbase.

We are planning to update HMRbase using above models at regular interval of three months.
